# Supplementary material for: Evaluation of Cookies Enriched with Osmodehydrated Wild Garlic from Nutritional and Sensory Aspects
Source: Foods. 2024 Jun 20;13(12):1941. doi: 10.3390/foods13121941 (PMC11202563; doi:10.3390/foods13121941)
Supplement: Supplementary file 1 [file foods-13-01941-s001.zip › foods-3005999-supplementary.pdf]

Supplementary S1.

Information for descriptive sensory analysis assessors

Information for the participants in the sensory analysis (assessors) regarding raw material content of the cookies enriched with osmodehydrated wild garlic:

- Whole spelt flour, manufacturer: “Jevtić”, Bačko Gradište, Serbia;
- Margarine, manufacturer: "Diamant", Zrenjanin, Serbia;
- Sugar, manufacturer: "Crvenka", Serbia;
- Table salt, manufacturer: "So produkt", Stara Pazova, Serbia;
- Baking soda ($NaHCO_3$) as the leavening agent, manufacturer: "Aleva", Novi Kneževac, Serbia;
- Oregano, manufacturer: "Aleva", Novi Kneževac, Serbia;
- Tap water, from the public water supply of Novi Sad, Serbia;
- Wild garlic, from forest area on the slopes of the Fruška Gora Mountain (45º08'34.6"N; 19º36'55.0"E), Serbia;
- Sugar beet molasses, used for osmotic dehydration process, maximal content in cookies' samples approx. 0.2%, manufacturer: "Crvenka", Serbia.

All materials used in cookies enriched with osmodehydrated wild garlic production are **edible food materials**.

Descriptive sensory analysis will be performed according to **following standards**:

- ISO 8586: 2012 General guideline for the selection, training and monitoring of selected assessors and expert sensory assessors;
- ISO 6658: 2017; Sensory Analysis—Methodology—General Guidance. ISO: Geneva, Switzerland, 2017;
- ISO 8589: 2007; Sensory Analysis—General Guidance for the Design of Test Rooms. ISO: Geneva, Switzerland, 2007;

and with the **strict adherence** to the ethical principles outlined in the **Declaration of Helsinki**.

Participation in the descriptive sensory analysis is voluntary, and assessors are in no obligation to perform descriptive sensory analysis, if do not agree with any of provided information.

Cookies samples descriptive sensory analysis form is provided in the appendix to this information.

Authors of the study

Cookies samples descriptive sensory analysis form

Sample no:												
**Attribute**			9 point intensity scale (1 - not detected; 9 - stongly present)									
**(D1) Apperance**	(D11)	Standard	1	2	3	4	5	6	7	8	9	
	(D12)	Colour Intensity	1	2	3	4	5	6	7	8	9	
	(D13)	Colour Uniformity	1	2	3	4	5	6	7	8	9	
	(D14)	Surface apperance	1	2	3	4	5	6	7	8	9	
**(D2) Taste**	(D21)	Bitter	1	2	3	4	5	6	7	8	9	
	(D22)	Spicy	1	2	3	4	5	6	7	8	9	
	(D23)	Caramel	1	2	3	4	5	6	7	8	9	
	(D24)	Herb	1	2	3	4	5	6	7	8	9	
**(D3) Odour**	(D31)	Pungent	1	2	3	4	5	6	7	8	9	
	(D32)	Caramel	1	2	3	4	5	6	7	8	9	
	(D33)	Spicy	1	2	3	4	5	6	7	8	9	
	(D34)	Herb	1	2	3	4	5	6	7	8	9	
**(D4) Texture**	(D41)	Hardness	1	2	3	4	5	6	7	8	9	
	(D42)	Fracturability	1	2	3	4	5	6	7	8	9	
	(D43)	Brittleness	1	2	3	4	5	6	7	8	9	
	(D44)	Mastication	1	2	3	4	5	6	7	8	9	

Supplementary S2

Z-Score Analysis

The maximal total Z-score values presents the optimum value of all segment Z-scores combined in appropriate manner, pointing at the optimal total quality of cookies' samples.

In the following equations the calculation of individual segment Z-scores is described:

Total polyphenol content and antioxidative activity and, segment Z-score:

$$S_{1i} = \frac{\sum_{k=1}^{3}\left(\frac{x_{ki}-x_{kmin}}{x_{kmax}-x_{kmin}}\right)+\sum_{j=1}^{2}\left(1-\frac{x_{ji}-x_{jmin}}{x_{jmax}-x_{jmin}}\right)}{5} \quad (1)$$

where x_k are: Total content of phenolics (TCP), Total content of flavonoids (TCF) and Total content of thiosulfates (TCT); and x_j are: Antioxidative activity by ABTS method (ABTS) and Antioxidative activity by DPPH method (DPPH).

Individual phenol compounds segment score:

$$S_{2i} = \frac{\sum_{l=1}^{6}\left(\frac{x_{li}-x_{lmin}}{x_{lmax}-x_{lmin}}\right)}{6} \quad (2)$$

where x_l are: Catechin, Chlorogenic acid, Kaempferol der.1, Kaempferol der.3, Kaempferol der.4 and Ferulic acid.

Dietary fibres and digestibility segment score:

$$S_{3i} = \frac{\sum_{m=1}^{4}\left(\frac{x_{mi}-x_{mmin}}{x_{mmax}-x_{mmin}}\right)}{4} \quad (3)$$

where x_m are: Betaine content, Total dietary fibres (TDF), Insoluble dietary fibres (IDF) and Digestibility.

Descriptive sensory analysis of appearance segment score:

$$S_{4i} = \frac{\sum_{n=1}^{3}\left(\frac{x_{ni}-x_{nmin}}{x_{nmax}-x_{nmin}}\right)+\left(1-\frac{x_{oi}-x_{omin}}{x_{omax}-x_{omin}}\right)}{4} \quad (4)$$

where x_n are: Standard appearance, Color intensity and Color uniformity; and x_o is Surface appearance.

Descriptive sensory analysis of taste segment score:

$$S_{5i} = \frac{\left(1-\frac{x_{pi}-x_{pmin}}{x_{pmax}-x_{pmin}}\right)+\sum_{r=1}^{3}\left(\frac{x_{ri}-x_{rmin}}{x_{rmax}-x_{rmin}}\right)}{4} \quad (5)$$

where x_p is: Bitter taste; and x_r are Spicy taste, Caramel taste and Herb taste.

Descriptive sensory analysis of odor segment score:

$$S_{6i} = \frac{\left(1-\frac{x_{qi}-x_{qmin}}{x_{qmax}-x_{qmin}}\right)+\sum_{s=1}^{3}\left(\frac{x_{si}-x_{smin}}{x_{smax}-x_{smin}}\right)}{4} \quad (6)$$

where x_q is: Pungent odor; an dx_s are Caramel odor, Spicy odor and Herb odor.
Descriptive sensory analysis of texture segment score:

$$S_{7i} = \frac{\sum_{t=1}^{3}\left(1-\frac{x_{ti}-x_{tmin}}{x_{tmax}-x_{tmin}}\right)+\left(\frac{x_{ui}-x_{umin}}{x_{umax}-x_{umin}}\right)}{4} \quad (7)$$

where x_t are: Hardness, Fracturability and Brittleness; and x_u is Mastication.
Total quality cookies samples' Z-score:

$$S_i = 0.2 \cdot S_{1_i} + 0.10 \cdot S_{2_i} + 0.2 \cdot S_{3_i} + 0.10 \cdot S_{4_i} + 0.20 \cdot S_{5_i} + 0.1 \cdot S_{6_i} + 0.1 \cdot S_{7_i} \quad (8)$$

where cookie samples' Nutritive quality characteristics Z-scores values (S_{1i} to S_{3i}) and Descriptive sensory analysis results Z-scores values (S_{4i} to S_{7i}) both represent 50% of total Z-score, or total quality.

$$\max [S_i] \rightarrow \text{optimum} \quad (9)$$
